# Continuous robust sound event classification using time-frequency features and deep learning

**DOI:** 10.1371/journal.pone.0182309

**Published:** 2017-09-11

**Authors:** Ian McLoughlin, Haomin Zhang, Zhipeng Xie, Yan Song, Wei Xiao, Huy Phan

**Affiliations:** 1 School of Computing, The University of Kent, Medway, Kent, United Kingdom; 2 National Engineering Laboratory of Speech and Language Information Processing, The University of Science and Technology of China, Hefei, PR China; 3 European Research Center, Huawei Technologies Duesseldorf GmbH, Munich, Germany; 4 The Institute for Signal Processing, University of Lübeck, Lübeck, Germany; Universidade de Vigo, SPAIN

## Abstract

The automatic detection and recognition of sound events by computers is a requirement for a number of emerging sensing and human computer interaction technologies. Recent advances in this field have been achieved by machine learning classifiers working in conjunction with time-frequency feature representations. This combination has achieved excellent accuracy for classification of discrete sounds. The ability to recognise sounds under real-world noisy conditions, called robust sound event classification, is an especially challenging task that has attracted recent research attention. Another aspect of real-word conditions is the classification of continuous, occluded or overlapping sounds, rather than classification of short isolated sound recordings. This paper addresses the classification of noise-corrupted, occluded, overlapped, continuous sound recordings. It first proposes a standard evaluation task for such sounds based upon a common existing method for evaluating isolated sound classification. It then benchmarks several high performing isolated sound classifiers to operate with continuous sound data by incorporating an energy-based event detection front end. Results are reported for each tested system using the new task, to provide the first analysis of their performance for continuous sound event detection. In addition it proposes and evaluates a novel Bayesian-inspired front end for the segmentation and detection of continuous sound recordings prior to classification.

## Introduction

Sound event classification requires a trained system, when presented with an unknown sound, to correctly identify the class of that sound. Robust sound event classification specifically introduces real-world complications into the classification task, most notably interfering acoustic noise, sounds occluded by overlap and event detection. Recent years have seen a significant amount of research into automatic sound classification, part of the greater research field now known as machine hearing [1]. In fact, a myriad of techniques and methods have been used for sound event detection and classification including automatic speech recognition (ASR) inspired methods [2, 3], signal processing-based approaches [4–6] and statistical classifiers [7, 8]. Many of these methods make use of mel-frequency cepstral coefficients (MFCCs) [9] or similar representations derived from ASR. More recent alternative features that have shown promise are those based on two-dimensional time-frequency representations such as the spectrogram image feature (SIF) [10–15] and stabilised auditory image (SAI) [14, 16, 17].

An important point to note is that of the 17 references cited above, only around half consider the effect of acoustic noise on the machine hearing task and only two [11, 13] specifically investigate overlapping or occluded sounds; the remainder consider only isolated sounds. Almost all, including those of the current authors [14, 15] are evaluated by classifying a database of sound files (usually one sound per file, and which may or may not have noise added), rather than a continuous recording of multiple, noisy and occluded sounds, which the current authors consider to be a more realistic scenario for actual deployment.

### Contribution

This paper adapts several state-of-the-art machine hearing methods into the classification of continuous, noise-corrupted and occluded sounds. It defines a first standardised evaluation method for such sounds, based on the commonly-used robust sound event classification evaluation task from [10–15] into a test that includes all three aspects of real-world performance; noise robustness, occlusion/overlap and event occurrence detection.

In this paper, we extend and evaluate several classifiers that have performed extremely well for the classification of isolated sound files. Isolated sound classification is a simpler task than continuous classification in that it firstly guarantees that each tested recording contains a sound to be detected, and secondly that only one sound is present.

Continuous classification, by contrast, may contain periods of time when no sounds are present, as well as times when one sound is present or when two or more sounds are overlapped. The continuous evaluation task incorporates all of these elements, and thus the classifiers need to be modified to account each of those cases, particularly in distinguishing between the no-sound and sound-present cases.

Having proposed an evaluation task, this paper develops continuous sound event detectors. We specifically begin with previously published isolated event classifiers that have demonstrated good performance as our baseline, namely MFCC with HMM [13], SIF with SVM [14], SIF with DNN [14] and SIF with CNN [15].

These will all be evaluated with an energy-based sound event detector front end which will be discussed below. Then, we will introduce and evaluate a novel sound event detector based on Bayesian Information Criteria (BIC) segmentation [18, 19], specifically for the CNN classifier. This will be shown to achieve excellent performance, although no attempt has been made to tune the operating parameters which have been set to match those of the best performing baseline systems.

#### Motivation

Machine hearing [1] describes the automated computer understanding of sound environments, just as machine vision is concerned with the automated understanding of visual information. Machine hearing is crucial for natural audio interfacing between humans and computers in diverse real world environments, and has particular application for speech interaction systems. Applications beyond this will have impact in fields such as security monitoring of homes and offices, environmental noise pollution and activity monitoring, and in enabling smart homes, buildings and cities.

As an example, in smart cities or in automated surveillance of public spaces, a computer could infer events from audible information using audio sensors that are lower cost, require less networking bandwidth, consume less power, are potentially more robust and less easily obscured by weather, dust or pollution than video sensors. They also have the ability to sense non-line-of-sight events and are likely to enjoy a lower computational burden for automated processing than moving image data. When used in a future smart city environment, networked audio sensors could be deployed city-wide at relatively low cost. At the very least, automated audio event detection could alert city staff to view appropriate video footage, at best it could trigger automated responses appropriate to the inferred events. The same is true of smart-home environments, or in security monitoring. As a human-computer interfacing aid, machine hearing allows a speech-based dialogue system to react to auditory events in a similar way to humans. Reactions could range from pausing dialogue in response to sounds, repeating words obscured by sounds as well as appropriate reaction to sounds as diverse as alarms, laughter, sneezes, screams, smashing glass, dog barks and car horns. In fact there are many identifiable everyday sounds that, during a conversation, one would normally expect both conversing parties to react to. For truly natural speech dialogue between human and computer, the computer should be expected to react to similar events as a human, and this implies machine hearing capabilities.

## Continuous robust audio event detection task

### The evaluation task

The evaluation task used in this paper builds upon the standard isolated sound evaluation task first reported by Dennis et.al. [12, 13]. The advantage of having a standard evaluation is that it is repeatable by others, and eases the comparison of results when other authors make use of the same method to evaluate their research [11, 14, 15]. The task uses freely available sound recordings from the Real World Computing Partnership (RWCP) Sound Scene Database in Real Acoustic Environments [20], with robustness evaluation performed by mixing these sounds with background noises from the NOISEX-92 database at several signal-to-noise (SNR) levels.

For the ‘traditional’ isolated sound evaluation, 50 sound classes, each comprising 80 recordings, are selected from the RWCP database. All sounds have both lead-in and lead-out silence sections and have no added noise. For each class, 50 randomly-selected files are used for training, with the remaining 30 reserved for evaluation. When cross-verifying, different selections of files are made. The arrangement and procedure for the isolated sound evaluation task can be found at http://www.lintech.org/machine_hearing with baseline code at [31].

Evaluation is performed separately and reported separately for clean sounds and those corrupted by additive noise. Noise-corrupted tests use four background noise environments selected from the NOISEX-92 database, namely “Destroyer Control Room”, “Speech Babble”, “Factory Floor 1” and “Jet Cockpit 1”. These environments were chosen as described by Dennis [12] to be realistic examples of non-stationary noise with predominantly low-frequency components.

To evaluate noisy conditions, one of the four NOISEX-92 recordings is randomly selected, a random starting point identified within the noise file, and then sample-wise added to the sound file. SNR is calculated over the entire noise and sound file in each case, and four separate test databases are created for clean sounds (i.e. no added noise), as well as noise mixtures with SNRs of 20, 10 and 0 dB.

For evaluation of continuous robust audio event detection, a new standard task is defined using the same auditory data as discussed above. Specifically, 100 separate 60 second sound vectors are created. 15 randomly selected instances from the 1500 test files (i.e. 30 examples from 50 classes) are then added into each sound vector at random positions. Finally, background noise is added in the normal way at the specified SNRs.

**Fig 1:**
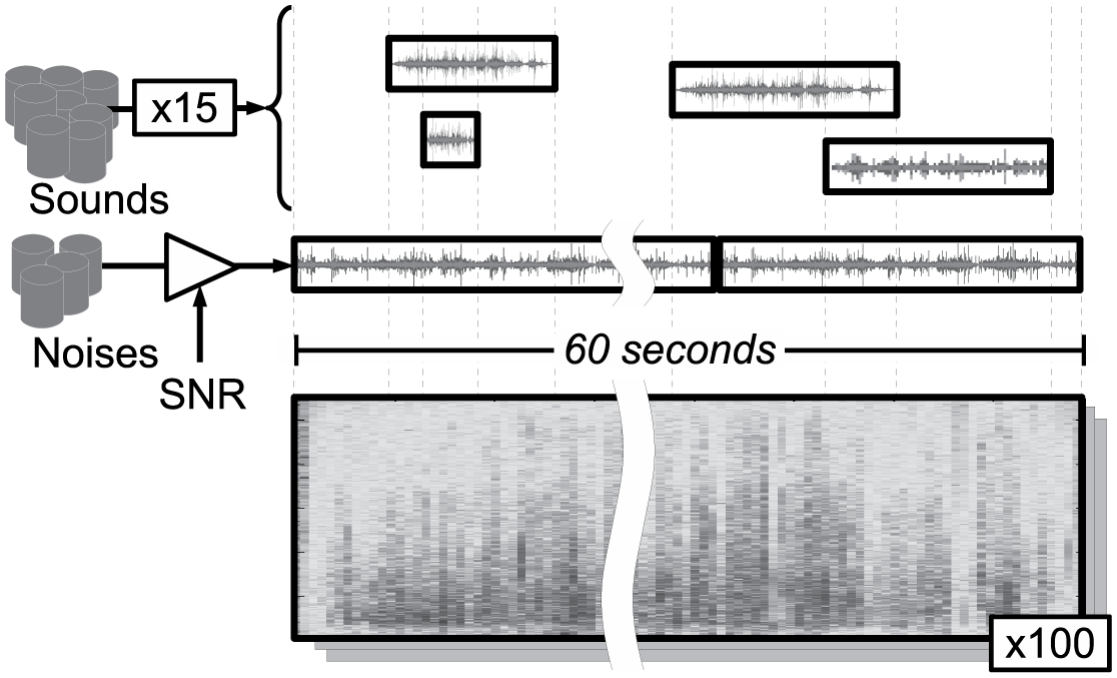
Diagram showing the construction of evaluation files containing overlapping occurrences of source sounds.

**Fig 2:**
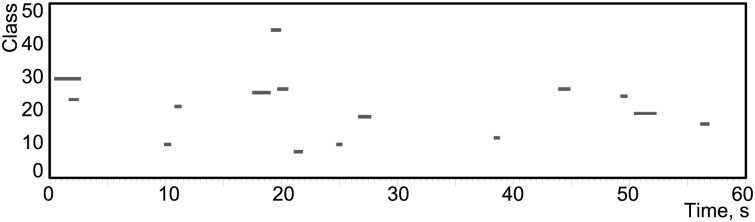
Illustration of one of the 60 second long evaluation recordings, containing a random selection of 15 different length recordings from the 50 classes.

There are thus four testing databases (clean, 20, 10 and 0 dB) each comprising a set of 100 different 60 s evaluation recordings. This process is illustrated in Fig 1, while a visualisation of one of the 100 recordings generated through this process is given in Fig 2, showing the times during which each of the 15 randomly selected sounds (chosen from the 50 classes) are present within the recording.

All of the test parameters and settings are summarised in Table 1, and the details of the files and steps required to create the test databases have been published and are available at http://dx.doi.org/10.17504/protocols.io.iw5cfg6.

**Table 1:** Testing and training datasets.

Parameter	value
Sound source	RWCP Sound Scene Database
Sound classes	50 impulsive sounds
Training sounds	50, randomly selected
Testing sounds	30, randomly selected
Noise source	NOISEX-92
Noise types	4 named files
Noise SNR	clean, 20 dB, 10 dB, 0 dB
Continuous file duration	60 s
Number of sounds per file	15, randomly placed
Number of test files	100
Total test duration	6,000 s
Tested sounds	1,500

Performance is assessed in terms of precision and recall. Precision *P* computes the proportion of all detected sounds that are of the correct class. This score evaluates how accurate the classification decisions are, but does not evaluate the performance of the detection process since it does not account for sound events that were not detected (and hence not classified). Recall *R*, by contrast, computes the proportion of detected sound events out of the total number of sound events. As is common in the literature, we make use of an F-measure to combine these, *F*_1_ = 2(*P*^−1^ + *R*^−1^)^−1^, and will use this in particular to explore trade-offs between precision and recall.

## Classifiers

This section will separately describe the following classifiers; MFCC with HMM [13] and then SIF with SVM [14], SIF with DNN [14], SIF with CNN [15] using energy-based event detection criteria. Finally, the Bayesian Inference Criteria (BIC) segmentation detector will be described.

### MFCC-HMM

MFCC features are extracted from 10 ms analysis frames with a 50% overlap. The first 12 MFCCs are concatenated with their frame-wise differential (Δ) and second differential (ΔΔ). A separate hidden Markov model (HMM) is then trained for each class in the evaluation data set. For continuous sound testing, the Viterbi algorithm is used to explore all possible state sequences to decode the observed test file feature sequences, obtaining the most probable model explanation.

### SIF with SVM, DNN and CNN

**Fig 3:**
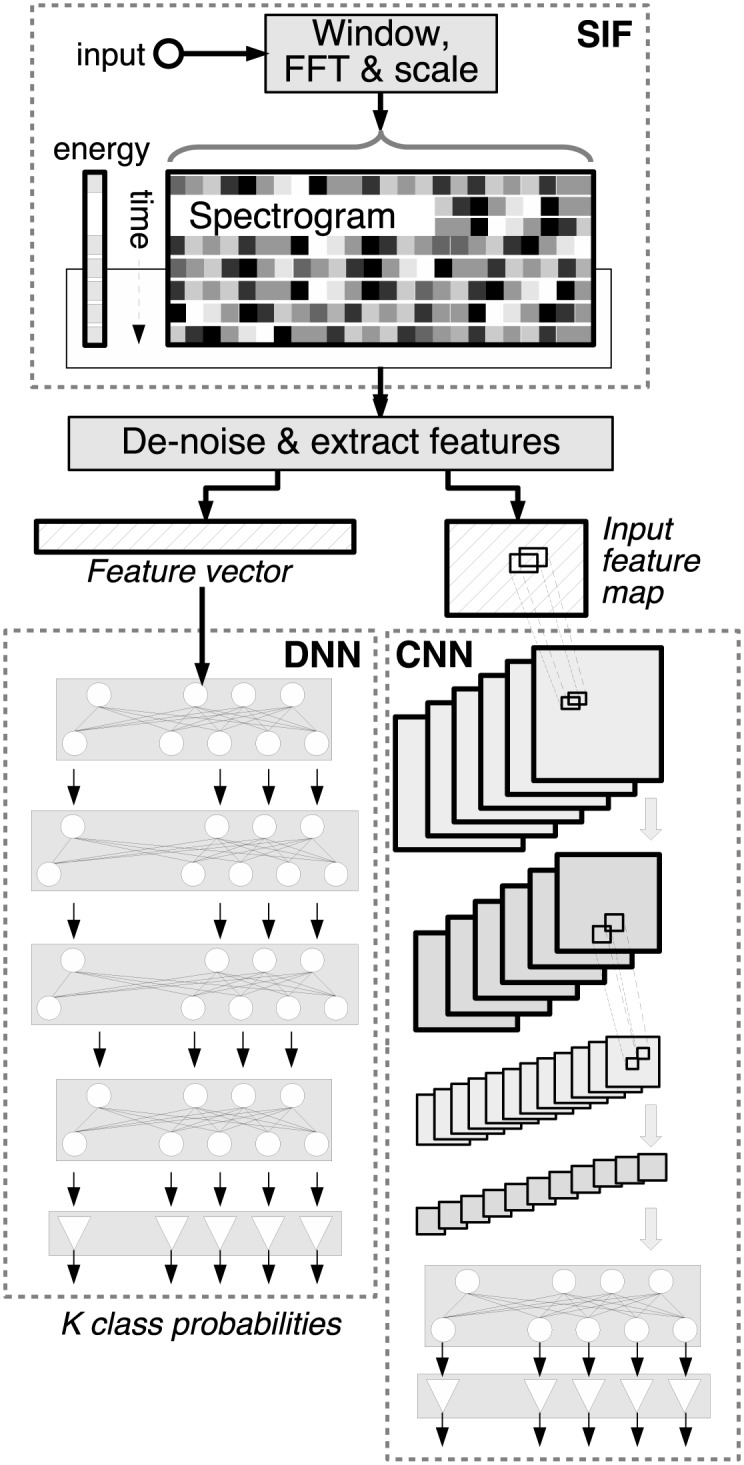
Block diagram of the SIF feature formation being used by a DNN and a CNN classifier.

This section describes the spectrogram image feature (SIF) as used with the various classifiers. The structure of the feature extraction and classification stages are compared in Fig 3, in particular for the DNN and CNN [14, 15]. The diagram shows the formation of the spectrogram and energy information into a matrix which is denoised and then formed into features. The DNN feature vector is formed from a rectangular region that is reshaped into a vector prior to classification by the DNN on the left, and is identical to that used in the SVM system (not shown). The CNN classifier on the right preserves the rectangular shape of the region as its input feature map. In each case, the classifier output is a set of *K* class probabilities. The energy and BIC detectors are used to select the time domain regions that form the input into Fig 3.

#### SIF

The SIF feature begins with a linear scaled and normalised spectrogram constructed from highly overlapped and windowed frames of length *w*_*s*_ samples. For frame index *F*, spectrogram *f*_*F*_(*k*) is obtained as follows from sound vector *s*_*F*_ which is extracted from sound file *s*,

$$\begin{array}{ccc} s_{F}\left(n\right) & = & s\left(F\delta w_{s}+n\right)w\left(n\right)\;for\;n=0\ldots w_{s}-1 \end{array}$$ (1)

$$\begin{array}{ccc} f_{F}\left(k\right) & = & |\sum_{n=0}^{w_{s}-1}s_{F}\left(n\right)e^{-j2\pi nk/w_{s}}|\;for\;k=0\ldots w_{s}-1 \end{array}$$ (2)

where *δ* is the advance between frames, in samples, *w*(*n*) defines a *w*_*s*_-point Hamming window. Spectrogram *f*_*F*_(*k*) is then downsampled in frequency into *B* bins by averaging over *B*′ = ⌊*w*_*s*_/2*B*⌋ samples. The resulting average spectra are then stacked to form an overlapped spectrogram (S),

$$\begin{array}{c} S\left(l,m\right)=\frac{1}{B^{\prime }}\sum_{n=lB^{\prime }}^{\left(l+1\right)B^{\prime }}f_{F-m}\left(n\right) \end{array}$$ (3)

To provide context, a history of up to *D* consecutive spectral lines (i.e. m=0…D-1) are concatenated to populate a *BD* + 1 dimension feature vector *V* which is augmented by a scalar energy measure, one per frame. Feature vector **v** comprises elements *v*(*i*);

$$\begin{array}{c} v(i)=S(\lfloor i/B\rfloor ,i-B\lfloor i/B\rfloor ))\;for\;i=0\ldots BD-1 \end{array}$$ (4)

with the scalar energy metric defined as;

$$\begin{array}{c} v\left(BD\right)=\sum_{l=0}^{D-1}\sum_{m=0}^{B-1}S\left(l,m\right) \end{array}$$ (5)

This captures frame energy, which is useful based on the hypothesis that very low energy frames are likely to be less discriminative to sound classification than higher energy frames. **v** is thus the input to the classifier feature extraction stage, with a dimensionality of only *DB* + 1.

In practice, several values of *B*, *D* and *δ* were tested and subsequently fixed to a system which balances efficiency with consistent performance, having *B* = 24, *D* = 30 and *δ* = 16. Each SIF analysis frame spans 16ms time duration with an 8 ms overlap between frames, and thus we observe that this method primarily operates by classifying short-time spectral characteristics. The final image dimensionality is thus *DB* + 1 = 721.

#### SVM

An input feature vector is denoted **v** = [*v*_1_, *v*_2_, …, *v*_*V*_]^⊤^, with length *V* and with **v** ∈ *R*^*V*^. This is to be classified into *K* classes, **y** = [*y*_1_, *y*_2_, …, *y*_*K*_]^⊤^, where **y** ∈ {1, −1}^*K*^. With a linear kernel, SVM solves the primal optimisation of the normal vector to the hyperplane, *w*;

$$\begin{array}{c} \min_{w,b,\xi }\frac{1}{2}w^{T}w+c\sum_{i=1}^{V}\xi_{i} \end{array}$$ (6)

*ξ* are slack variables which are used to define an acceptable tolerance, and *c* > 0 is a regularisation constant. *ψ*(**v**_*i*_) maps **v**_*i*_ to a higher dimensionality, and,

$$\begin{array}{c} y_{i}\left(w^{T}\psi \left(v_{i}\right)+b\right)\geq 1-\xi_{i},\;\xi_{i}\geq 0,\;i=1\ldots V \end{array}$$ (7)

Since **w** typically has high dimensionality [21], for computational efficiency we usually solve the related problem,

$$\begin{array}{c} \min_{\alpha }\frac{1}{2}\alpha^{T}Q\alpha +e^{T}\alpha \end{array}$$ (8)

with **e** = [1, …*V*]^*T*^ being a vector of all ones. Q is a positive semi-definite matrix of dimension *V* × *V* with *Q*_*ij*_ ≡ *y*_*i*_ *y*_*j*_ *K*(**v**_*i*_,**v**_*j*_). The kernel function *K*(**v**_*i*_,**v**_*j*_) ≡ *ψ*(**v**_*i*_)^*T*^ *ψ*(**v**_*j*_) is linear in this instance. Eq (8) is subject to the constraint **y**^*T*^ **α** = 0, 0 ≤ **α**_*i*_ ≤ *c*, for *i* = 1…*V*.

Having solved Eq (8), using the primal-dual relationship, the optimal **w** satisfies,

$$\begin{array}{c} w=\sum_{i=1}^{V}y_{i}\alpha_{i}\psi \left(x_{i}\right) \end{array}$$ (9)

and the decision function becomes the sign of **w**^*T*^ *ψ*(**v**_*i*_) + *b* from Eq (7) which is easily computed from,

$$\begin{array}{c} sgn(\sum_{i=1}^{V}y_{i}\alpha_{i}K\left(v_{1},v\right)+b) \end{array}$$ (10)

The SVM input feature vector was scaled and mapped to a [−1, +1] input range prior to training and testing using *v*(*i*) = {*u*(*i*) − min(**u**)}/{(max(**u**) − min(**u**))} for *i* = 1…*V*, where *u*(*i*) denotes the *i*th element of unscaled input vector **u** and *v*(*i*) represents the *i*th element of the scaled feature vector **v**.

This is implemented using LIBSVM [21] with which alternative kernels are easily evaluated. Tested kernels were linear $K\left(v_{i},v_{j}\right)=v_{i}^{T}v_{j}$, third order polynomial $K\left(v_{i},v_{j}\right)={\left(\gamma v_{i}^{T}v_{j}\right)}^{3}$, radial basis *K*(**v**_*i*_,**v**_*j*_) = *e*^−*γ*||**v**_*i*_ − **v**_*j*_||^2^^ and sigmoid $K\left(v_{i},v_{j}\right)=\tanh\left(\gamma v_{i}^{T}v_{j}\right)$.

**SVM system parameters:** Development testing revealed that best performance was achieved overall using a linear kernel $v_{i}^{T}v_{j}$ with regularisation constant *c* = 32. *γ* was estimated by the LIBSVM toolkit and set to 0.03. This is close to the default (i.e. 1/*N* = 0.02) but resulted in slightly improved performance. All parameters were fixed globally (i.e. maintained as constant for all classes) over the *K*(*K* − 1)/2 binary models required to partition the results into *K* classes using one-against-one models. Majority voting was applied to contiguous frames to determine overall classification score for a particular region.

We evaluated systems with 50 and 51 classes. The latter reserved a single class for ‘no sound’ analysis frames, however performance was found to be very poor, most likely due to the lack of a positive energy signal to discriminate against (i.e. the classifier was effectively being trained on the absence of something rather the presence of something). Thus, the systems evaluated in this paper have 1225 binary classifiers yielding *K* = 50 class outputs.

#### DNN

We constructed an *L*-layer deep neural network (DNN) with the input fed from the chosen feature vectors (e.g. SIF, shown in Fig 3) and the output layer in a one-of-*K* configuration (given *K* classes) The DNN begins with a number of individually pre-trained restricted Boltzmann machine (RBM) pairs, each of which have *V* visible input nodes and *H* hidden stochastic nodes, **v** = [*v*_1_: *v*_*V*_]^⊤^, and **h** = [*h*_1_: *h*_*H*_]^⊤^ which are then stacked to form a deep network. The DNN input layer is formed from a Gaussian-Bernoulli RBM with real input nodes **v**_*gb*_ ∈ *R*^*V*^ and binary hidden nodes **h**_*gb*_ ∈ {0, 1}^*H*^, whereas inner layers are Bernoulli-Bernoulli having binary visible and hidden nodes, **v**_*bb*_ ∈ {0, 1}^*V*^ and **h**_*bb*_ ∈ {0, 1}^*H*^.

Let *w*_*ji*_ represent the weight between the *i*th visible and the *j*th hidden unit, so that weight matrix **W** = {*w*_*ij*_}_*V*×*H*_. Let $b_{i}^{v}$ and $b_{j}^{h}$ represent the respective real-valued biases, such that $b^{h}={\left[b_{1}^{h},b_{2}^{h},...,b_{H}^{h}\right]}^{⊤}$ and $b^{v}={\left[b_{1}^{v},b_{2}^{v},...,b_{V}^{v}\right]}^{⊤}$. In a Gaussian-Bernoulli RBM, every visible unit *v*_*i*_ adds a parabolic offset to the energy function, governed by *σ*_*i*_, which is generally predetermined, rather than derived from the data. The Gaussian-Bernoulli RBM energy function can be described [22] as,

$$\begin{array}{c} E_{gb}\left(v,h\right)=-\sum_{i=1}^{V}\sum_{j=1}^{H}\frac{v_{i}}{\sigma_{i}}h_{j}w_{ji}+\sum_{i=1}^{V}\frac{{\left(v_{i}-b_{i}^{v}\right)}^{2}}{2\sigma_{i}^{2}}-\sum_{j=1}^{H}h_{j}b_{j}^{h} \end{array}$$ (11)

The Gaussian-Bernoulli RBM model parameters are thus *θ*_*gb*_ = {**W**, **b**^**h**^, **b**^**v**^, *σ*^2^}. The energy function of the Bernoulli-Bernoulli RBM for state *E*_*bb*_(**v**, **h**) is computed similarly, but does not require *σ*_*i*_ given the binary nature of input nodes,

$$\begin{array}{c} E_{bb}\left(v,h\right)=-\sum_{i=1}^{V}\sum_{j=1}^{H}v_{i}h_{j}w_{ji}-\sum_{i=1}^{V}v_{i}b_{i}^{v}-\sum_{j=1}^{H}h_{j}b_{j}^{h} \end{array}$$ (12)

Bernoulli-Bernoulli RBM model parameters are thus *θ*_*bb*_ = {**W**, **b**^**h**^, **b**^**v**^}. Given an energy function *E*(**v**,**h**) defined as in either Eq (11) or Eq (12), the joint probability associated with configuration (**v**, **h**) is defined as,

$$\begin{array}{c} p\left(v,h;\theta \right)=\frac{1}{Z}e^{\left\{-E(v,h;\theta )\right\}} \end{array}$$ (13)

where *Z* is a partition function, *Z* = ∑_**v**_∑_**h**_ *e*^{−*E*(**v**,**h**;*θ*)}^.

**Pre-training:** RBM model parameters *θ* are typically estimated from training data in a maximum likelihood sense using contrastive divergence (CD) [23]. This algorithm updates hidden nodes **h** by stepping through a Gibbs Markov chain with early termination, given visible nodes **v** and previously updated **h**. Layer 1 hidden nodes are trained first based on the input feature vector (from training data). The states of the trained hidden units then become the visible data for training layer 2, and the process repeats to produce multiple trained layers of RBMs. These are then stacked to produce the DNN.

**Fine-tuning:** A softmax output labelling layer of *K* units is appended to the pre-trained stack of RBMs [24]. The function of the layer is to convert the Bernoulli distributed outputs in the final layer into a multinomial distribution. If *p*(*k*|**h**_**L**_; *θ*_*L*_) is the probability of the DNN classifying final output layer states **h**_**L**_ into the *k*-th class then,

$$\begin{array}{c} p\left(k|h_{L};\theta_{L}\right)=\frac{e^{\sum_{i=1}^{H}w_{ki}h_{i}+b_{k}}}{\sum_{p=1}^{K}e^{\sum_{i=1}^{H}w_{pi}h_{i}+b_{p}}} \end{array}$$ (14)

where $\theta_{L}=\left\{\theta_{gb}^{1},\theta_{bb}^{2}...\theta_{bb}^{L}\right\}$ are the trained model parameters for the entire *L*-layer DNN. Back propagation (BP) is then used to train the stacked network, including the softmax class layer, based on minimising the cross entropy error, $C=-\sum_{k=1}^{K}c_{k}\log p(k|h;\theta_{L})$, between the true class label, *c* and that predicted by the softmax layer.

**DNN system parameters:** The DNN classifier is implemented using the winning structure as defined in the authors’ previous work [14], which is a five layer network of the form 721 − 210 − 210 − 50 with dropout during training (the proportion of weights fixed during each training batch, in order to prevent over-training) of 0.1, mini-batch training size of 100 and up to 1000 training epochs. Momentum is 0 and learning rate begins at 10 then drops to 5 after 100 epochs, 2 after 400 and 1 after 800 [14].

As with SVM, the DNN classifier has 50 output classes, one for each sound. Again, the benefit of an additional ‘no sound’ class was explored and found to be detrimental in practice. The consequence of this is that the DNN (and SVM) systems are forced to assign each analysis frame to one of 50 classes, with no way to indicate absence of sound, i.e. they are doing sound classification rather than sound detection. Thus a separate means of detecting the absence or presence of sound is necessary. In general, two methods are described in this paper, the first being a short-time energy detector described in the following subsection and the second being a novel BIC method discussed later.

### CNN

Convolutional neural networks (CNNs) are multi-layer neural networks typically consisting of several pairs of convolution layers and subsampling layers plus a set of fully connected output layers. While the large number of layers and degree of connectivity describes a network that is high in complexity, weights are shared within layers to reducing the number of parameters that require training. Despite this simplification, CNNs share the need for relatively large amounts of training data with DNNs, and yet have been shown to outperform DNNs in several fields including image processing [25, 26] and ASR [27, 28].

A spectrogram of sound events is essentially an image of different time-frequency patterns, many of which exhibit local relationships but only weak absolute locality, i.e. recognisable sounds may appear at different times and in slightly different frequency ranges. CNNs have been shown able to classify image data well [25, 26] and are insensitive to pattern placement within an image (thanks to the convolution and subsampling steps), thus are potentially well-suited to sound event classification from two dimensional time-frequency spectrogram input. In this application, the CNN feature map is constructed from spectrogram and energy information as shown in Fig 3.

As with multi-layer perceptrons (MLPs), CNNs can be trained by gradient descent using back-propagation. Since units in the same feature map share the same parameters, the gradient of a shared weight is simply computed as the sum of the shared parameter gradients.

In general, for a convolutional layer *l*, we form the *j*th output map $x_{j}^{l}$ from

$$\begin{array}{c} x_{j}^{l}=f\left(\sum_{i\in M_{j}}x_{i}^{l-1}*k_{\text{ij}}^{l}+b_{j}^{l}\right), \end{array}$$ (15)

where $x_{i}^{l-1}$ is the *i*th input map, $k_{\text{ij}}^{l}$ denotes the kernel that is applied, and *M*_*j*_ is one of a selection of input maps [29]. The subsampling layer is simpler, $x_{j}^{l}=f\left(\beta_{j}^{l}↓\left(x_{i}^{l-1}\right)+b_{j}^{l}\right)$ with ↓(.) representing sub-sampling and *β* and *b* being biases. After repeating convolutional and subsamping layer pairs, the output is formed by what is effectively a dual layer (or deeper) MLP. The size of the MLP input layer is determined by the total number of nodes in the final CNN subsampling layer, while the size of the MLP output layer is determined by the number of classes.

**CNN system parameters:** The CNN classifier is implemented based on the method presented in [15], except that the classification is performed on all detected energy points rather than just three per file. Each energy point triggers a set of six overlapping analysis frames that are downsampled to a resolution of 52 × 40 and then fed to the input layer of the CNN. The five layers comprising the CNN then consist of a 5 × 5 kernel convolution layer with outputmap size 6 followed by a 2 : 1 subsampling layer, then a second 5 × 5 kernel convolution later with outputmap size 12 and a final 2 : 1 subsampling layer. The output layer feeds a two-layer fully interconnected MLP that has 50 output classes, yielding *K* output probabilies as per Eq (14).

### Energy detector

The energy detector uses both instantaneous peak energy and short-time energy criteria to detect candidate frames for sound classification. Specifically, if *E*_*F*_ is the energy of frame *F*, then if *E*_*F*_ > *ϵ* and *E*_*F*_ > *E*_*F*−*i*_ : *E*_*F*+*i*_ where *i* = −2*D*…2*D*, the current frame and its context is selected for classification.

For the experimental results presented in this paper, the threshold is simply set to the mean energy of all *N*_*F*_ frames, i.e. $\epsilon =\frac{1}{N_{F}}\sum_{N_{F}}E_{F}$ (where *E*_*F*_ has been pre-calculated as *v*(*BD*) for SIF features), leading to a large number of potential trigger positions, limited only by the temporal criteria.

If an experimental evaluation comprises *N*_*F*_ analysis frames in total, the effect of the energy detector is to reduce the number of frames to be classified to $N_{F}^{\prime }$ where $N_{F}^{\prime }<N_{F}$. This means that the array of features, originally of dimension [*BD* + 1, *N*_*F*_] is then reduced to dimension $\left[BD+1,N_{F}^{\prime }\right]$ prior to classification. The classifier will then output dimension $\left[K,N_{F}^{\prime }\right]$ classification probabilities.

### Bayesian inference detector

The BIC approach attempts to partition an input array into two parts that have more similar statistical distributions *within* each part than *between* parts. Given a search window *z*, which we construct from contiguous features, two hypotheses are considered. *H*_0_ is that *z* is distributed according to a single Gaussian model, and *H*_1_ is that *z* is distributed according to two Gaussian models and can thus be separated into two different models *x* and *y* [30]. We next define,

$$\begin{array}{ccc} \Delta BIC & = & BIC\left(H_{1}\right)-BIC\left(H_{0}\right)\\ & = & Nlog|\Sigma_{z}|-\frac{1}{2}\lambda \left(d+d\left(d+1\right)/2\right)logN\\ & - & N_{y}log|\Sigma_{y}|-N_{x}log\left|\Sigma_{x}\right| \end{array}$$ (16)

where *N*, *N*_*x*_ and *N*_*y*_ = *N* − *N*_*x*_ are the window lengths of models *z*, *x*, and *y*, *d* is the feature dimension and Σ_*z*_, Σ_*x*_, Σ_*y*_ are covariance matrices of the feature estimates from each respective window. For the results presented here, we use a fixed model complexity penalty *λ* = 1.0, and model the Gaussians on 39 dimension features comprising MFCC, ΔMFCC and ΔΔMFCC, computed frame-wise [30].

We exhaustively compute Δ*BIC* for all possible partitionings within the set. In each case, if max(Δ*BIC*) > 0, then hypothesis *H*_1_ is true and *t* = argmax(Δ*BIC*) marks a separation point whereas if max(Δ*BIC*) < = 0, then hypothesis *H*_0_ is true and there is no partition in window *z*. The process repeats, iteratively splitting windows until either all remaining windows are best represented by a single Gaussian distribution, or the length of a remaining window is smaller than the minimum allowed for classification. In practice, *z* spans 200 overlapping SIF analysis windows with a very large overlap of 199 (i.e. 16 ms) so that initial BIC segment sizes are 1.608 s in duration. Each split window is then subjected to the energy detector as usual, to obtain a detection point (with their usual backwards-forward context) within each window. This implies at least one classification result for every window, meaning that every BIC error automatically contributes a classification error.

As with the energy detector, the Bayesian inference detector similarly reduces the number of frames of features for classification. We can again denote this as having dimension $\left[BD+1,N_{F}^{\prime }\right]$ prior to classification, although the number and identity of frames chosen using the two detection methods will of course differ.

### Background probability scaling and thresholding

When either the energy or BIC detectors are used, the result is a sequence of $N_{F}^{\prime }$ candidate frames for classification, that are input to the feature extraction block, shown in Fig 3. Each frame, *F* is classified separately by the DNN or CNN to derive a set of posterior probabilities, *p*(*k*|**θ**) for trained model **θ** from Eq (14) where *k* = 1…*K*.

Contemporary sound classification algorithms tend to expect isolated sound events, typically arranged with one sound occurrence per file Given *N*_*F*_ analysis frames in a recording, each classified separately, the overall classification is computed by looking at all classes over all *N*_*F*_ frames. Either the posterior probabilities for each class are simply summed over all frames to find the class with highest aggregate score, or the probabilities are first scaled by the frame energy prior to summation [14]. Neither method works well for continuous sounds, due to the uncertainty regarding start and end positions of sounds and the case where no sounds are present but the classifiers are forced to choose. Certain classes are inherently more noise-like, so that classifying NOISEX-92 background noise in the absence of foreground sounds results in persistent misclassifications into a small number of classes. It is thus necessary to normalise the output probabilities.

Given classification probability *p*(*k*, *n*) for class *k* in frame *n*, we obtain the long term average classifier output probability over *N*_*F*_ frames, $\bar{p}\left(k\right)=\frac{1}{N_{F}}\sum_{n=1}^{N_{F}}p\left(k,n\right)$ for all classes *k* = 1…*K*. Now, instead of attributing each frame to ${arg max}_{k}p(k,n)$ and then attributing the entire recording to the class which wins the highest number of frames as in non-continuous systems [14], we will instead determine the winning class for each classification region as the one that has the highest probability compared to the mean posterior probability;

$$\begin{array}{c} max\left(p\left(k,n\right)-\chi \left\{\bar{p}\left(k\right)-\sum_{K}\bar{p}\left(k\right)/K\right\}\right)>p_{TH}\;\text{for}\;k=1\ldots K,n=1\ldots N_{F}^{\prime } \end{array}$$ (17)

where *χ* accounts for the degree to which background noise triggers individual classifiers. Testing trained classifiers in the presence of noise alone, reveals that several sound classes have an inherent similarity to some periods of background noise. In a system which classifies a segment of audio based directly on the highest posterior probability, noise is therefore often miss-attributed to noise-like classes, causing miss-classification. However the difference between actual sounds and background noise is twofold. Firstly, actual sounds cause continuously high probabilities from their matching class, whereas spurious noise triggers are sporadic and usually of much shorter duration. Secondly, actual sounds—even in high levels of noise—exhibit a higher probability score from their matching class compared to the background probabilities by other classes. We thus introduced *p*_*TH*_ as a probability threshold that balances the trade-off between false-positive and false-negative classifications and *χ* to account for background noise triggering. In practice a *χ* of 0.2 was sufficient to prevent background noise triggering, and this was fixed for the remaining tests. The probability threshold *p*_*TH*_ is then varied to plot receiver operating curves (ROC), allowing us to explore the performance of different detectors. Neither parameter is tuned independently for each tested system, as discussed below, however it is expected that careful adjustment of *p*_*TH*_ using a development data set would yield optimal values for each system.

## Results and discussion

This section will first present the performance of each of the classifiers and features for the ‘traditional’ task of classifying isolated sound files according to the standard evaluation task, then evaluate the same classifiers for continuous classification. We will explore the baseline classification using an energy detector, then evaluate the use of probability scaling and thresholding, both with and without the BIC detector. Finally, we will explore the influence of the probability threshold *p*_*TH*_ on performance.

### Baseline isolated sound results

Table 2 presents the classification accuracy by HMM with MFCC features, and by SVM, DNN and CNN using SIF features. The systems are each evaluated in different levels of NOISEX-92 background noise. The mean result is computed over all noise conditions to provide a single measure of the performance of each system for comparison. From these results it is clear that MFCC-HMM performs best in noise-free conditions (‘clean’), but degrades rapidly with increasing acoustic noise. None of the SIF-based methods perform quite as well as MFCC-HMM in noise-free conditions, but all are able to maintain performance with only small degradation as noise levels increase. The ASR-inspired MFCC-HMM method is thus the least noise-robust method, while SIF-CNN appears most capable for the 20 dB and 10 dB conditions, which are likely to encompass the main range of realistic deployment scenarios, while SVM maintains a slight advantage in the highly noisy 0 dB environment.

**Table 2:** Classification accuracy for the four implemented continuous sound event detection methods in different levels of SNR.

System	clean	20dB	10dB	0dB	mean
MFCC-HMM	99.47	54.00	21.27	05.67	45.10
SIF-SVM	96.40	96.27	95.60	87.13	93.85
SIF-DNN	92.47	92.07	91.33	79.87	88.94
SIF-CNN	97.27	97.20	96.13	85.67	94.07

By mean performance, the SIF-CNN system performs best, followed by SIF-SVM and then SIF-DNN. The comparatively good performance of the CNN classifier in noise echoes the results of other research [15].

### Continuous sound results

Having established an isolated sound classification benchmark for each of these systems, we now aim to evaluate performance for the continuous task, however we first perform a series of experiments to assess the trade-off between recall and precision achieved by adjusting the probability threshold *p*_*TH*_.

Results shown in Table 3 are the recall, precision and *F*_1_ score for the mean performance over all noise types (i.e. clean, 20 dB, 10 dB and 0 dB SNR) for three systems, and for a range of *p*_*TH*_ settings.

**Table 3:** Precision, recall and *F*_1_ for CNN classifier using energy detector and BIC, respectively, for feature selection over a range of different probability thresholds.

System	*p*_*TH*_:	0.9	0.8	0.7	0.5	0.3	0.1
SIF-CNN/Baseline	Precision	92.2	84.6	78.8	68.7	65.0	64.8
	Recall	62.7	71.3	75.8	80.9	82.3	82.3
	*F*_1_	74.7	77.3	77.3	74.3	72.6	72.5
SIF-CNN/prob. scale	Precision	95.0	90.0	83.9	72.1	65.4	64.7
	Recall	60.4	70.0	75.5	80.7	82.3	82.3
	*F*_1_	73.8	78.7	79.5	76.2	72.9	72.4
SIF-CNN/BIC	Precision	96.5	94.0	91.2	86.7	78.4	77.7
	Recall	57.4	66.5	71.5	75.0	78.1	78.1
	*F*_1_	72.0	77.9	80.2	80.4	78.2	77.9

The first system is a straightforward implementation of the SIF-CNN baseline system using an energy detector to trigger classification regions and a majority vote of classifier outputs. We can see that the best *F*_1_ is achieved when *p*_*TH*_ is 0.8 or 0.7, however precision is maximised at a higher *p*_*TH*_ and recall is maximised at a lower threshold.

The second system applies the background probability scaling and thresholding methods, such that the classification outputs within a detection region are normalised with respect to the mean classification output probabilities as discussed above. The effect of this is to improve the peak *F*_1_ score, and slightly increase precision, at the expense of recall. This is to be expected because it will naturally result in more selective classification regions (hence increasing precision), at the expense of additional false negatives (hence affecting recall). Again the best *F*_1_ score is achieved at a *p*_*TH*_ of around 0.7 to 0.8, whereas the best precision and recall are at the extremes of the table. Clearly, the *p*_*TH*_ setting is operating as a tradeoff between the two conflicting demands of better recall and better precision.

The final system uses the BIC separation method at the front-end prior to the energy detector and probability scaling/thresholding. The results reveal that the optimum *p*_*TH*_ for overall *F*_1_ score is now lower at about 0.5. Interestingly, while precision has improved substantially over other methods, recall is slightly degraded. The final combined *F*_1_ score achieves over 80% accuracy.

Table 4 now presents results for continuous detection and classification for several systems in different levels of noise, with overall with *p*_*TH*_ fixed to 0.7. According to the results in Table 3, *p*_*TH*_ = 0.7 was the best value for the baseline system but is slightly sub-optimal for the proposed SIF-CNN/BIC method. Further experimentation using a development data set would be required to determine an optimal *p*_*TH*_ for each system, and this may reasonably be expected to further enhance the SIF-CNN/BIC results. In the following section, different *p*_*TH*_ settings will be evaluated to determine a receiver operating curve (ROC) response.

**Table 4:** Mean precision, recall and *F*_1_ score achieved by the implemented systems on the continuous task under various noise conditions.

System	Precision					Recall					*F*_1_
SNR	clean	20dB	10dB	0dB	mean	clean	20dB	10dB	0dB	mean	mean
MFCC-HMM	28.12	08.69	06.60	04.57	12.00	94.87	79.20	60.47	38.53	68.27	20.41
SIF-SVM	90.84	85.87	57.32	27.51	65.39	86.93	86.80	85.60	71.20	82.63	73.01
SIF-DNN	87.70	82.53	53.69	24.63	62.14	84.87	84.33	81.33	64.13	78.67	69.43
SIF-CNN/Baseline	93.66	92.03	77.99	51.67	78.84	81.80	81.67	79.33	60.47	75.82	77.30
SIF-CNN/BIC	95.79	94.95	89.67	84.40	91.20	76.67	77.73	75.53	56.20	71.53	80.18

The results in Table 4 show that all of the tested deep neural learning systems outperform the HMM in all but the recall of clean sounds (a task at which the MFCC-HMM system excels with almost 95% performance). This confirms results for isolated sound classification systems reported elsewhere [13, 14].

The results also confirm the good performance of CNNs, especially for the important noise-corrupted tests. More surprisingly, SVM performance is highly competitive to the CNN system in all cases, more so than the DNN in fact. When comparing these results to the isolated sound classification performance, it appears that the SVM classifier is better able to accomplish detection (i.e. distinguishing presence versus absence of sound) than the CNN. Contrasting the SIF-CNN and SIF-CNN/BIC results, it seems that the BIC segmentation method performs better than the energy detector in general, apart from slightly lower recall due to the more selective nature of the segmentation. The proposed SIF-CNN/BIC system achieves the best combined *F*_1_ score, as well as the best precision for all noise conditions. Comparing the continuous classification precision to the isolated sound recognition accuracy, it is notable that apart from the MFCC-HMM system, the evaluated techniques degrade by less than 10% in accuracy for clean sounds, but by as much as 50 to 60% at 0dB SNR. The implication is that the detection process is less noise robust than the underlying classification process.

**Fig 4:**
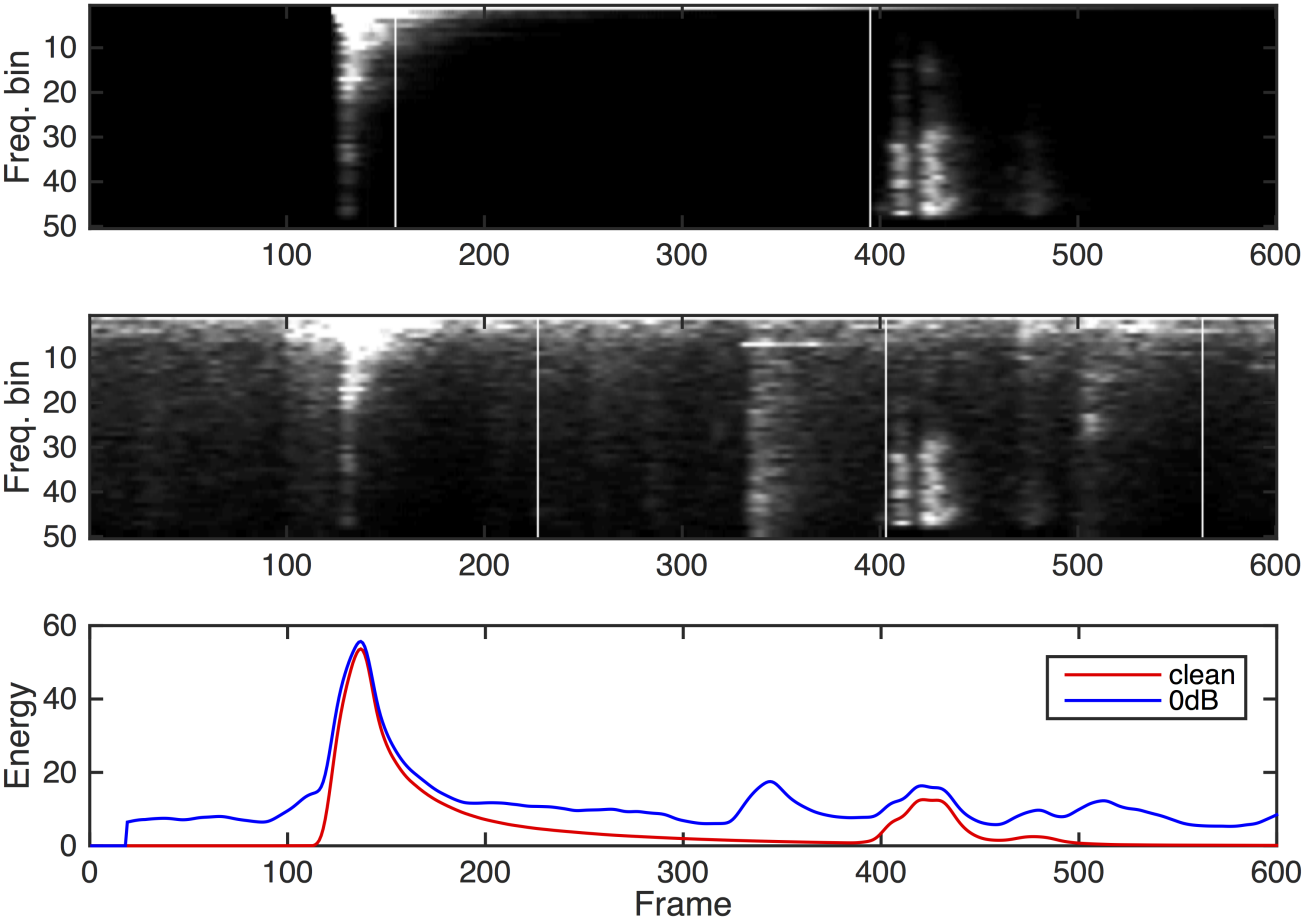
Spectrograms of two sounds combined into one file without added noise (top), in the presence of 0 dB noise (middle), and frame-wise energy plots (bottom). Vertical lines are BIC segmentation markers.

To better visualise the process, Fig 4 plots spectrograms of a 9.6s long segment of one test recording. The upper spectrogram is without additional noise, whereas the one below it is the same region with noise added at an SNR of 0 dB. For clarity, this segment only contains two sounds, and these are visible not only in the spectrograms but also in the frame-by-frame energy plot. Vertical lines in the spectrogram are drawn to indicate BIC segmentation markers in each case, with more segmentations occurring in the noisy case.

**Fig 5:**
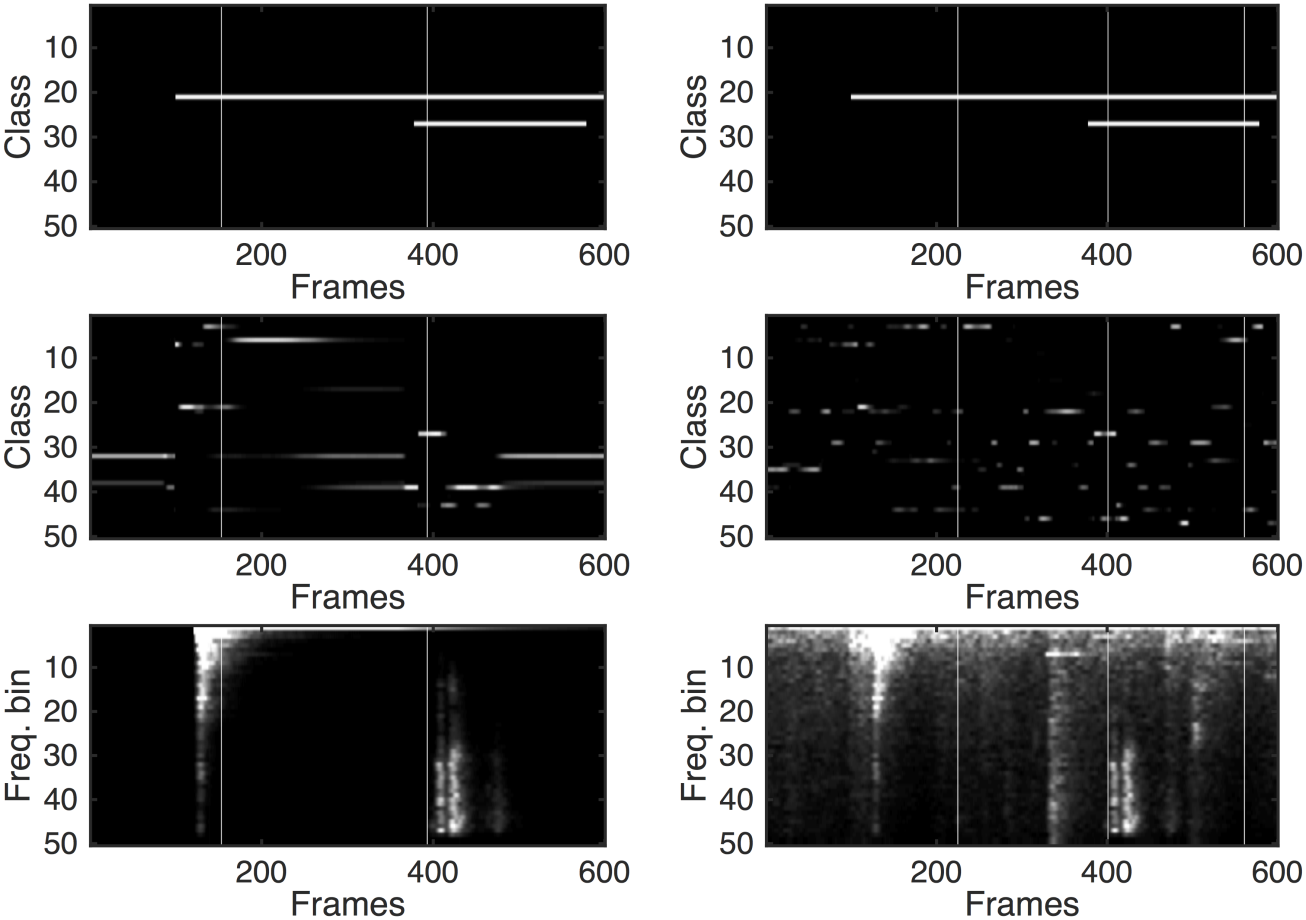
Clean (left) and 0 dB (right) plots showing actual classes present (top), classifier output probabilities (middle) and spectrograms (bottom). As in Fig 4, the vertical lines indicate BIC segmentation markers.

To explore further, Fig 5 uses the same example to visualise the classification probabilities. The figure shows the actual sound classes that are present (top), the classifier output probabilities (middle) and re-plots the corresponding spectrograms (bottom). The noisy example (right hand side) evidently exhibits far more spurious classification points than the clean recording (left hand side) but in both cases, several classes are continuously active. The influence of these is countered by the background probability scaling and thresholding process.

### Probability threshold and tradeoffs

**Fig 6:**
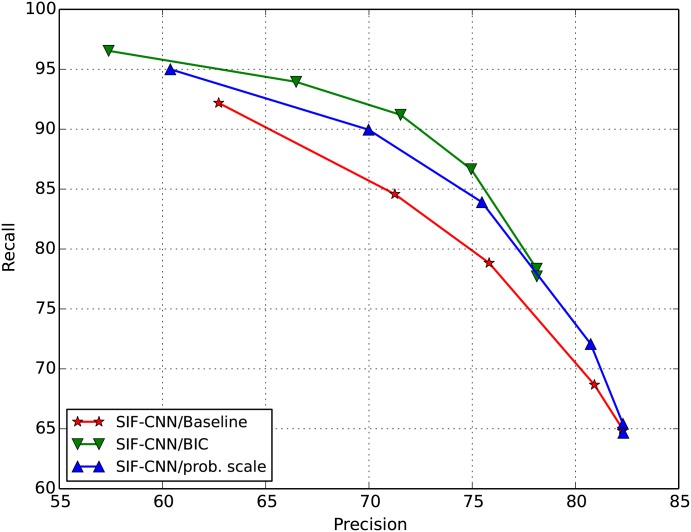
Receiver operating curves for three detection methods.

Fig 6 displays an ROC plot of recall against precision for the three systems, namely the SIF-CNN baseline, probability scaled and BIC methods. Each of these are evaluated in terms of mean *F*_1_ score over all noise conditions. This evaluation is performed for a range of probability thresholds to adjust the trade-off points between recall and precision. What is clear from the graph is that the background probability scaled system outperforms the baseline, and in turn the proposed SIF-CNN/BIC method outperforms the background probability scaled method.

## Conclusion and future work

Classification of sounds in potential future deployment scenarios will require robust approaches that work in the presence of interfering acoustic noise, with sounds that may be occluded or overlapping, and which can operate continuously with no prior knowledge of the start and end times of sounds. This paper has extended three state-of-the-art machine-learning based sound event classification methods to the continuous case: these methods have previously only been evaluated for classification of isolated sounds or those having known starting and ending times.

This paper has additionally proposed a standard evaluation task for overlapping continuous sounds, based upon the commonly-used evaluation task for isolated sounds. This has been used to evaluate the robustness of the various techniques. As other authors develop their own continuous sound event classification algorithms, it is hoped that they will adopt the same evaluation criteria, since it consists of easily available data, and presents a realistic deployment scenario.

In this paper, all evaluated methods use energy-based criteria to detect candidate onset positions for sounds, while a Bayesian inference criteria has been developed specifically for the CNN classifier, and shown to yield a performance improvement. Results show that classification performance reduces by an average of approximately 20 to 30% (in terms of precision) between the isolated and continuous cases, with by far the largest degradation occurring at the highest noise levels, implying that the detection process is inherently less noise robust than the classification process. Other researchers may therefore expect to obtain good performance gains in future by separating and separately optimising the detection and classification tasks, and by exploring the effect of tuning parameters such as *p*_*TH*_, *χ*, *λ*, *ϵ*, as well as the number of CNN layers, outputmap and subsampling parameters.
